# Methodological considerations for measuring glucocorticoid metabolites in feathers

**DOI:** 10.1093/conphys/cow020

**Published:** 2016-06-10

**Authors:** Sara A. Berk, Julie R. McGettrick, Warren K. Hansen, Creagh W. Breuner

**Affiliations:** 1 Organismal Biology and Ecology, University of Montana, Missoula, MT 59812, USA; 2 Department of Chemistry and Biochemistry, University of Montana, Missoula, MT 59812, USA; 3 Wildlife Biology Program, University of Montana, Missoula, MT 59812, USA

**Keywords:** Corticosterone, feather, glucocorticoid, metabolite, stress

## Abstract

In recent years, researchers have begun to use corticosteroid metabolites in feathers (fCORT) as a metric of stress physiology in birds. However, there remain substantial questions about how to measure fCORT most accurately. Notably, small samples contain artificially high amounts of fCORT per millimetre of feather (the small sample artefact). Furthermore, it appears that fCORT is correlated with circulating plasma corticosterone only when levels are artificially elevated by the use of corticosterone implants. Here, we used several approaches to address current methodological issues with the measurement of fCORT. First, we verified that the small sample artefact exists across species and feather types. Second, we attempted to correct for this effect by increasing the amount of methanol relative to the amount of feather during extraction. We consistently detected more fCORT per millimetre or per milligram of feather in small samples than in large samples even when we adjusted methanol:feather concentrations. We also used high-performance liquid chromatography to identify hormone metabolites present in feathers and measured the reactivity of these metabolites against the most commonly used antibody for measuring fCORT. We verified that our antibody is mainly identifying corticosterone (CORT) in feathers, but other metabolites have significant cross-reactivity. Lastly, we measured faecal glucocorticoid metabolites in house sparrows and correlated these measurements with corticosteroid metabolites deposited in concurrently grown feathers; we found no correlation between faecal glucocorticoid metabolites and fCORT. We suggest that researchers should be cautious in their interpretation of fCORT in wild birds and should seek alternative validation methods to examine species-specific relationships between environmental challenges and fCORT.

## Abstract (precis)

Stress hormone metabolite levels in feathers represent an integrated picture of hormone physiology during feather growth. We address several current methodological issues and provide suggestions for future research.

## Introduction

Animals release glucocorticoids in response to perceived challenge. In wild animals, these hormones are typically measured in the blood. However, researchers can turn to non-invasive metrics, such as hair, faeces or feathers, when capture and handling of an animal to obtain a blood sample is not feasible and/or desirable (Goymann *et al.*, 2002; Bortolotti *et al.*, 2008, 2009; Meyer and Novak, 2012). Plasma sampling gives a snapshot of the concentration of corticoterone (CORT) and its metabolites at the time of capture, but CORT deposited in other tissues represents hormone levels over a longer time period. By using alternative measures, researchers can gain a more integrated picture of physiology over a period of hours (faeces), weeks or months (feathers and hair). These newer methods have exciting potential for the field of conservation physiology, as evaluating physiology over longer time periods can help us to assess the effects of human-mediated environmental changes. Although these benefits are attractive, especially to the conservation researcher, we must take caution to measure glucocorticoids accurately in new tissues in order to avoid spurious or misleading results (Goymann, 2012).

Of the alternative measures listed above, extraction of corticosterone metabolites from feathers (hereafter, fCORT) is the most recently developed and least validated. Corticosterone (and its metabolites) are incorporated into the feather as it develops and are therefore thought to represent an integrated picture of physiology over the duration of feather growth (Bortolotti *et al.*, 2008, 2009; Jenni-Eiermann *et al.*, 2015). Researchers have been quick to apply fCORT to a variety of systems, from sexual selection to environmental enrichment (e.g. Fairhurst *et al*., 2011; Kennedy *et al*., 2013; Lendvai *et al*., 2013). Although these early studies provide promising results, some have noted substantial methodological issues. For example, Lattin *et al*. (2011) found that fCORT per milligram of feather increased exponentially with decreasing mass (which caused small samples to have artificially high fCORT per milligram of feather), creating a small sample artefact. Furthermore, many researchers have attempted to correlate fCORT and serum CORT and found mixed results. In red-legged partridges, fCORT and stress-induced serum CORT are positively correlated, although this relationship may be driven by two outliers (Bortolotti *et al.*, 2008). Studies that have used CORT implants have found that fCORT primarily reflects serum CORT when CORT is experimentally elevated (Fairhurst *et al.*, 2013; Jenni-Eiermann *et al*. 2015), and fCORT in birds with CORT implants are elevated even in sections of the feather that grow prior to implantation (Lattin *et al.*, 2011). A recent study also found that when plasma CORT was elevated by a food-deprivation treatment, birds changed their feather growth rate, resulting in less fCORT per millimetre of feather in food-deprived birds (Patterson *et al.*, 2015). Hence, measures of fCORT in feathers are more complex than researchers are currently recognizing. If we understand how fCORT relates to other measures of CORT physiology (plasma or faecal), we can better interpret correlations between fCORT and environmental conditions or performance. If fCORT does not represent natural circulating concentrations of hormone over the time the feather was grown, then correlations between fCORT and other variables may not be biologically relevant.

Although faecal hormone studies have validated the ability of various antibodies to detect CORT using high-performance liquid chromatography (HPLC; e.g. Wasser *et al.*, 2000), there has not been a similar push to measure antibody sensitivity for fCORT. Jenni-Eiermann *et al.* (2015) performed a successful validation of an enzyme immunoassay corticosterone kit, but most laboratories currently use radioimmunoassay and a CORT antibody from Sigma-Aldrich (Lattin *et al*., 2011). Previous work has confirmed the presence of corticosterone, cortisol and testosterone in feathers, but to date there has been no confirmation that the Sigma-Aldrich antibody is not binding to compounds in the feather besides corticosterone that would distort results (Rettenbacher *et al.*, 2009; Koren *et al.*, 2012).

Here, we used four approaches to address several issues related to the measurement of corticosteroid metabolites in feathers. First, we examined whether the fCORT small sample artefact identified by Lattin *et al*. (2011) in European starling feathers is a general problem across species. Then we tested whether this artefact resulted from a decrease in extraction efficiency with larger mass, as has been found in faecal samples. Hayward *et al*. (2010) were able to find a significant effect of experimental treatment (anthropogenic noise) only when they used a revised protocol that included adjustment of the amount of methanol relative to faecal mass during extraction, removal of urates, fully lyophilizing samples and using a more accurate scale. Therefore, we were interested in whether adjusting methanol would have a similar effect on fCORT to reduce the effect of small sample size on CORT extraction. Next, we compared fCORT with faecal glucocorticoid metabolites (FGMs) during feather growth. We expected that FGMs would more closely predict fCORT than plasma CORT because both fCORT and FGMs are integrated measures of glucocorticoid physiology over extended periods. We hoped to provide a biologically relevant link between fCORT and endogenous glucocorticoid physiology, currently almost entirely lacking in the literature (but see Bortolotti *et al*., 2008). Lastly, we used HPLC to identify steroid metabolites in feathers and characterized which compounds were bound by the commonly used Sigma-Aldrich antibody in the fCORT radioimmunoassay.

## Materials and methods

### Feather sources

Tree swallow (*Tachycineta bicolor*) and harlequin duck (*Histronicus histronicus*) feathers were collected through other projects studying reproductive effort and feather corticosterone at Beaverhill Lake, Alberta, Canada (2012) and Glacier National Park, MT,USA (2010–12), respectively. Tree swallows were captured at nest boxes, and ∼10 rump feathers were collected. Harlequin ducks were captured during pre-egg lay on the breeding stream, and during this capture we collected one or two tail feathers and one to four back feathers. Feathers were stored in envelopes until analysis. Sharp-tailed grouse (*Tymphanuchus phasianellus*) feathers were provided by local hunters from the Missoula, MT, USA area. Grouse were frozen whole; we pulled feathers from the wings and body as needed for assays. Feathers for the house sparrow assays were collected during the captive experiment described in Part III below. Two outer tail feathers were pulled and allowed to regrow during the experiment. Those regrown tail feathers were then assayed for fCORT. All animal handling procedures were approved by the University of Montana and Cornell University Institutional Animal Care and Use Committee (house sparrow, University of Montana AUP #015-14CBOBE-32814; harlequin duck, University of Montana AUP #011-11CBDBS-041311; and tree swallow, Cornell University AUP #2001-0051).

### Part I: demonstration of the small sample artefact across species

We manipulated the length of sharp-tailed grouse wing and body (back and rump) feathers to test the relationship between the amount of feather and picograms per millimetre (pg/mm) and picograms per milligram (pg/mg) fCORT extracted. The first assay included 24 grouse body feathers ranging in length from 10 to 74 mm. Within this group, 14 feathers were cut in intervals of 10 mm between 10 and 70 mm, while the remaining 10 samples included entire feathers not cut to a specific length so that they encompassed the natural variation for grouse body feathers (54–74 mm). For the adjusted-length feathers, we measured feathers from the tip and cut feathers to the desired length to avoid as much as possible the chance of irregular CORT deposition across the feather (Bortolotti *et al.*, 2009). The second assay included 26 flight feathers that ranged in length from 10 to 90 mm. All grouse feathers in this experiment were incubated in 7 ml methanol. We used feathers from two individual grouse, but each assay included samples from one only bird, so the two individuals were never compared against each other. Duck back and tail feathers were assayed whole (without the calamus as per Bortolotti *et al*., 2008; please see Hansen *et al*. (2016) for test of cut vs. whole feathers). The range of lengths and weights was provided by natural variation as well as by the numbers of feathers used (one or two tail feathers and one to four back feathers). All duck feathers were incubated in 5 ml methanol (Table 1). We chose harlequin ducks and sharp-tailed grouse to test the small sample artefact because we had large quantities of feathers from these species readily available in our laboratory.

**Table 1:** Description of feather assays, including the length and mass range of samples included

Species	Feather region	Length range (mm)	Mass range (mg)
Sharp-tailed grouse	Flight	10–90	3.6–79.7
Sharp-tailed grouse	Body	10–74	2.1–22.9
Harlequin duck	Tail	23–89	1.8–71.4
Harlequin duck	Back	16–54	1.0–22.0

### Part II: evaluation of methanol extraction efficiency

To test the possible effect of extraction efficiency on pg/mm or pg/mg fCORT extracted, we ran three separate assays with different ranges of millilitres of methanol/feather length or mass (hereafter, concentrations). First, we examined extraction efficiency against mass, using a pool of swallow rump feathers from 23 individuals that we cut to <5 mm^2^ and mixed thoroughly to control for within- and between-individual variation in CORT deposition (after Lattin *et al.*, 2011). We used this pool for the mass evaluation but used single feathers for length evaluation (Part I above). This is suboptimal because there may be within-individual variation in CORT deposition across feathers, but we were unable to make a feather pool that was standardized by length. We used concentrations between 0.1 and 10 ml methanol/mg feather across 26 samples that were weighed to either 1 or 5 mg. Next, we examined extraction efficiency against length (millilitres of methanol per millimetre of feather), by using two additional assays of grouse rump and back feathers from two individuals. For the first of these assays, we used 17 grouse flight feathers (from one individual) that we cut to either 10 or 20 mm and extracted at concentrations ranging from 0.33 to 2 ml methanol/mm feather. The second assay included 36 samples (rump and back feathers from one individual) with methanol/feather concentrations between 0.05 and 0.5 ml/mm and cut feathers to 10, 20 or 40 mm. Currently, researchers extract CORT from feathers at 0.25 (a 20 mg feather sample in 5 ml methanol) to 0.7 ml methanol/mg feather (a 10 mg sample in 7 ml methanol; Lattin *et al.*, 2011; Lendvai *et al.*, 2013; Kennedy *et al.*, 2013; Kouwenberg *et al.*, 2015; although many papers do not report the mass of feather samples), Therefore, we chose concentrations that were both close to and much higher or lower than this ratio to determine maximal extraction efficiency.

### Part III: correlation of faecal glucocorticoid metabolites and fCORT

We held 20 house sparrows in individual cages on natural day length with food and water *ad libitum* from March to May 2014. We ran this experiment in two sets, one containing eight individuals and lasting 26 days and the other containing 12 individuals and lasting 29 days. We collected faeces and urates from wax paper placed at the bottom of each cage. Samples collected within one 5 h period were combined and analysed as one sample per day of feather growth (resulting in 26 samples per individual for group 1 and 29 samples per individual for group 2). The first group of birds grew feathers that ranged in length from 27 to 40 mm (mean 33.2 mm, SD 3.93 mm), and the second group of birds grew feathers that ranged in length from 29 to 49 mm (mean 37.54 mm, SD 5.5 mm). In the first group, three individuals did not regrow rectrices during the experiment, so we were able to correlate feather and faecal CORT in only five individuals from this group. These birds that did not grow feathers during the experiment were released, and their data are not included here.

### Part IV: high-performance liquid chromatography

We analysed the corticosterone metabolites in grouse flight feathers using HPLC. Hormone metabolites were extracted with methanol (from four secondary feathers from one individual sharp-tailed grouse) and reconstituted in 1% acetic acid in methanol. We used an Agilent 1260 Infinity instrument equipped with a degasser, quaternary pump, autosampler and diode array detector. We carried out chromatographic separations on an Agilent Zorbax Eclipse C18 column (4.6 mm  × 100 mm, 3.5 µm). Each sample was eluted in a linear gradient between mobile phase A (methanol–water–acetic acid, 40 : 60 : 1 v/v/v) and B (methanol–water–acetic acid 60 : 40 : 1 v/v/v). The gradient started from 10% B and went to 40% B over 10 min, followed by a 5 min gradient to 50% B, then the column was eluted with 100% B for 5 min. The flow rate was 1.0 ml/min, and ultraviolet absorbance detection was done at 254 and 300 nm. Forty 500 µl fractions per feather sample were collected using a BioRad model 2110 fraction collector. We assessed immunoreactivity of these fractions against our Sigma-Aldrich antibody by combining fractions from the four feather samples that had been run individually through the HPLC (total = 2 ml per sample) before drying down with nitrogen and resuspending in a phosphate-buffered saline solution (PBSG). We then tested the immunoreactivity of each fraction using radioimmunoassay (described below).

To determine what metabolites were present, we ran standards through the HPLC both individually and together. We identified potential corticosterone and other steroid-family metabolites based on data from Miksik *et al*. (1999) and Lattin *et al*. (2011). All standards were purchased from Steraloids (Wilton, NH, USA). Although we began with 13 metabolites, we were able to achieve good separation and detection of 4-pregnen-11β,21-diol-3,20-dione (corticosterone); 4-pregnen-11β,21-diol-3,18,20-trione (progesterone metabolite); 4-pregnen-20β,21-diol-3,11-dione (progesterone metabolite); 4-pregnen-11β,20β,21-triol-3-one (20β-’dihydrocorticosterone); 5α-androstan-3α,11β-diol-one ’(testosterone metabolite); 1,3,5(10)-estratrien-3,17β-diol (estradiol); 5β-pregnan-3α,21-diol-11,20-dione (tetrahydro-11-dehydrocorticosterone); 5β-pregnan-3α,20β,21-’triol-11-one (progesterone metabolite); 4-pregnen-11β-17,’21-triol-3,20,dione (cortisol); and 4-pregnen-21-ol-3,20-dione hemisuccinate (deoxycorticosterone). We determined that a standard was identifiable using HPLC if that standard displayed a singular ultraviolet peak when analysed alone, and this peak was again present during analysis of all of the standards together. We attempted but were unable to achieve good detection of 5α-pregnan-3β, 11β,21-triol-20-one (progesterone metabolite); 4-pregnen-3,20-dione (progesterone); and 4-pregnen-21-ol-3,11,20-trione (11-dehydrocorticosterone).

### Hormone assays

We measured fCORT following Bortolotti *et al*. (2008). Briefly, feathers were cut to the appropriate length, weighed, and added to varying amounts of methanol (see Parts I and II, above; house sparrow feathers from the FGM and fCORT study were incubated in 7 ml methanol). All feather samples were cut to <5 mm length before extraction except for the duck feathers, which were run as part of a separate study. Samples were sonicated for 30 min and then incubated overnight in a 50°C water bath. The feather was separated from methanol with vacuum filtration, and methanol was evaporated in a 50°C water bath under nitrogen gas. Samples were reconstituted into PBSG (pH 7.0) and incubated overnight at 4°C. Hormone levels were measured in duplicate with radioimmunoassay following Wingfield *et al*. (1992) using the Sigma-Aldrich antibody (C 8784; Saint Louis, MO, USA). This antibody has been successful in measuring corticosterone and its metabolites in the past (see Bortolotti *et al*. 2008, 2009; Lattin *et al*. 2011). We determined recoveries for two out of five feather assays by spiking samples with a small amount of ^3^H-CORT. Recoveries ranged from 70 to 100% (average = 86%, SD = 10%). Although we spiked samples, we did not adjust based on recoveries in these two assays, so adjusted data were never statistically compared against non-recovery data. Goymann (2005) recommended against using recoveries when extracting CORT from non-plasma sources, noting that recoveries of pure CORT may not match recoveries of metabolized CORT and so are not necessarily helpful in determining extraction. Intra-assay variation averaged 3.8%.

Faeces were dried, sieved, and extracted in 20× methanol. All extracts were then subjected to the same protocol described above, with modifications as per Mauget *et al*. (1994). The FGM levels were determined using the Sigma-Aldrich antibody listed above, which has been successfully optimized and used in house sparrows in a previous study (Chávez-Zichinelli *et al*., 2010). We ran one to three individuals per assay, distributed over seven assays. Intra-assay variation was 6.1%, and inter-assay variation was 39%. Samples were therefore adjusted according to variation in the standard by multiplying them by the factor that the standard was different from the mean standard value across assays. Notably, results were similar with and without the correction, so we present uncorrected values here (see Results section).

### Statistical analyses

Analyses were run in Prism 6.0 (GraphPad Prism, San Diego, CA, USA) and R software packages (R Core Team 2014; available at http://www.R-project.org). For small sample artefact analyses, we fitted quadratic equations and compared these fits with a straight line using Akaike’s information criterion (AIC). To analyse the effect of methanol volume on CORT extraction, we used generalized linear models with an identity link function (one for each assay) to examine the effects of feather amount and methanol on total pg/mg or pg/mm CORT. We used a backwards model selection process that began with the interaction of feather mass or length group and methanol concentration. Results for this interaction are reported only if this term was significant.

Even though we collected faeces starting on the day of feather pulling, feather regrowth did not begin until several days later. We measured feathers after they emerged at 14, 17 and 27 days post feather-pull. We found that the slope of this line was consistent across individuals, and we were able to estimate feather emergence at 10 days post feather-pull for our first group of birds. For this reason, we removed all faecal samples prior to day 10 from our analyses. The relationship between FGMs and fCORT was assessed with a generalized linear model (with an identity link function) that included an effect of faecal sample mass. As faecal CORT data were skewed, we performed a log transformation prior to analysis. We tested for the effect of group (first or second) on FGM but found no support for this term (β = −0.3831, *P* = 0.43), so we did not include it in our final model. For comparisons of faecal and feather hormone concentrations, we scaled all variables (faecal pg/mg, feather pg/mm and faecal sample mass) to a mean of zero and a standard deviation of one.

## Results

### Part I: demonstration of the small sample artefact across species

**Figure 1:**
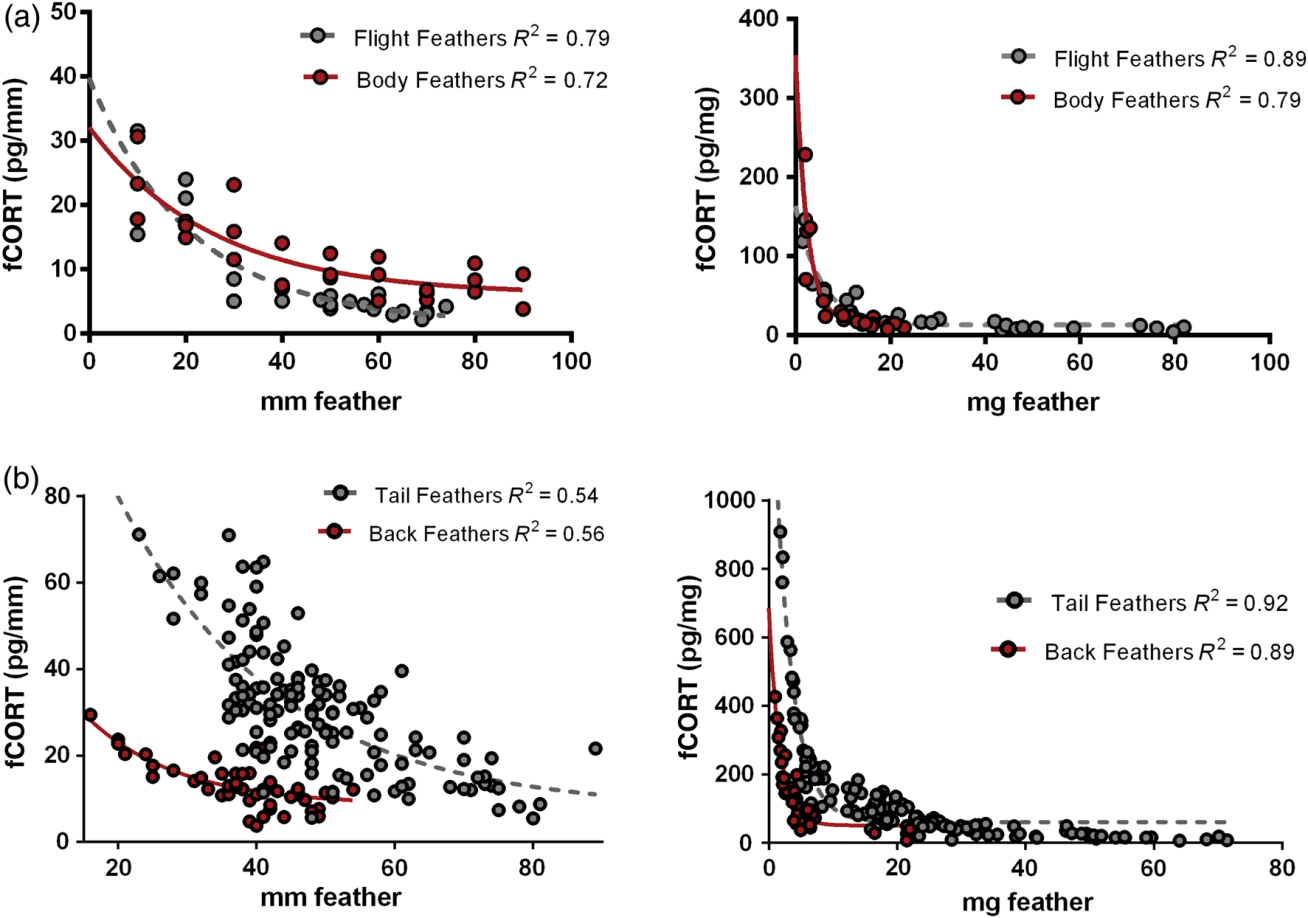
We found a strong effect of feather length and mass on picograms per millimetre or picograms per milligram corticosteroid metabolites in feathers (fCORT) in sharp-tailed grouse (**a**) and harlequin ducks (**b**). (a) Feather corticosteroid metabolites in grouse flight and body feathers expressed per millimetre (left) and per milligram (right). Both sharp-tailed grouse assays included feathers standardized to a range of lengths. (b) Feather corticosteroid metabolites in harlequin duck tail and back feathers from a separate assay, expressed per millimetre (left) and per milligram (right). The assays include whole harlequin duck feathers from both back and tail.

The fCORT per milligram and fCORT per millimetre increased as milligrams and millimetres of feather decreased across two feather types in two species. Thus, small samples had higher pg/mm or pg/mg fCORT than larger samples (Fig. 1). The data were best fitted by a quadratic equation (ΔAIC > 2 for each comparison) rather than a straight line.

### Part II: evaluation of methanol extraction efficiency

**Figure 2:**
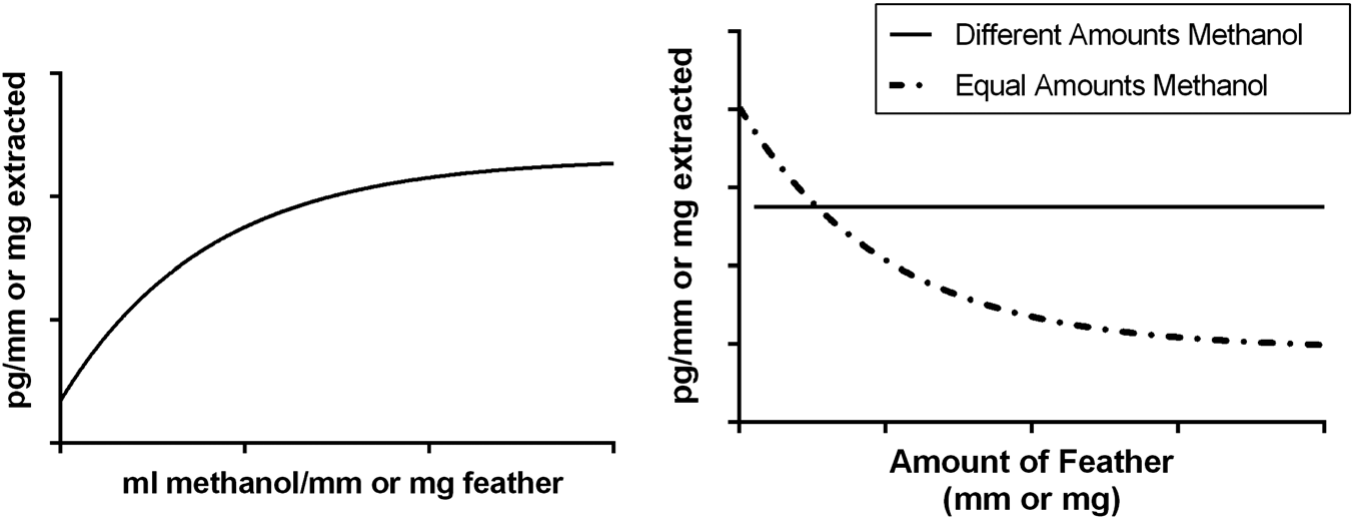
Hypothetical relationships if the relative amount of methanol to feather material can reduce small sample artefact. Left panel: picograms per millimetre or picograms per milligram feather should increase as more methanol relative to feather is added until all corticosteroid metabolites have been extracted from the feather. Right panel: if we adjust methanol in all samples to achieve maximal extraction, we should eliminate the small sample artefact (a current problem in feather hormone analysis, represented by the dot-dashed line), and instead see no relationship between the amount of feather material in each sample and corticosteroid metabolite extraction (continuous line).

**Figure 3:**
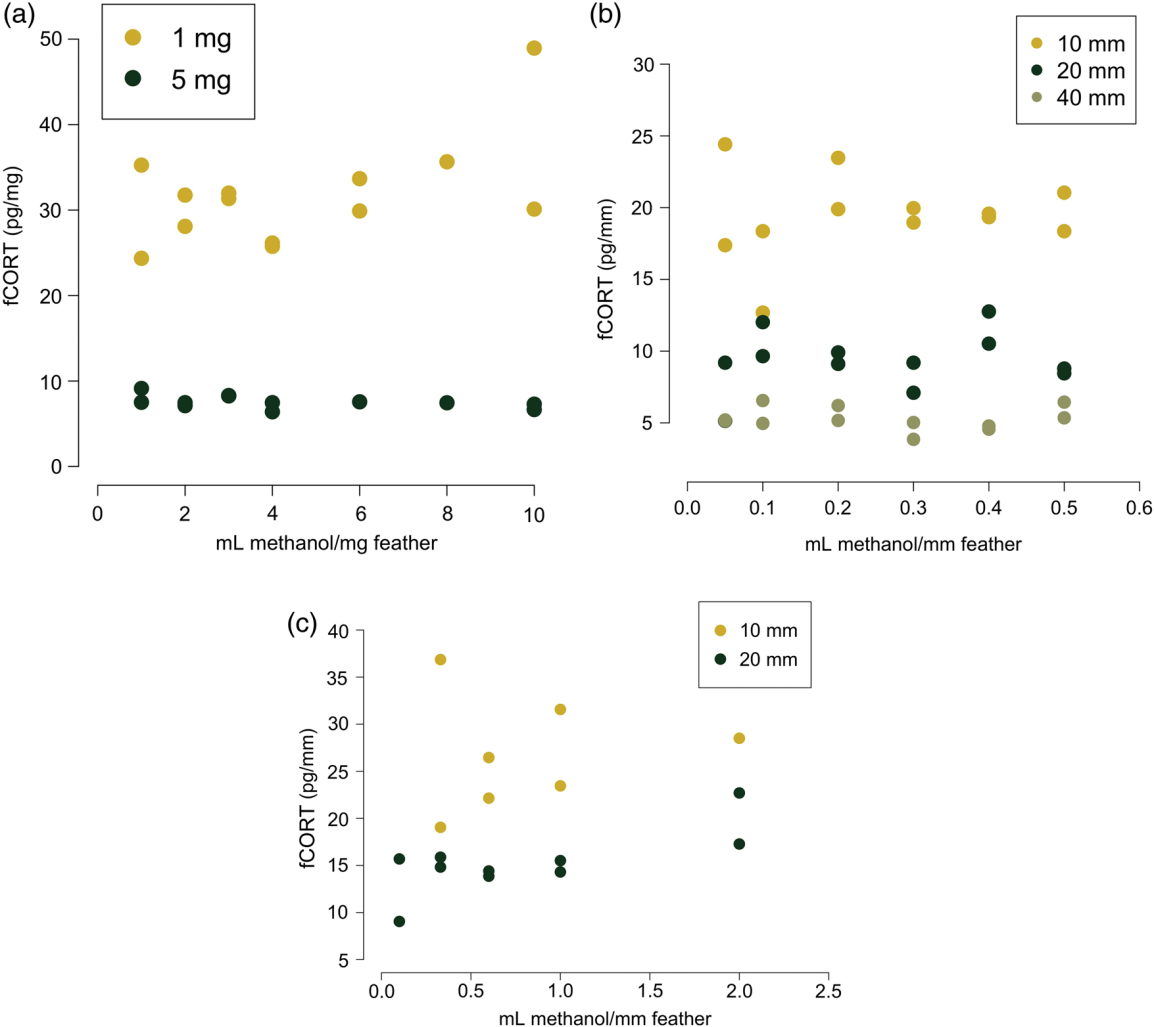
The relationship between the amount of methanol (in millilitres per millimetre or per milligram of feather) and corticosteroid metabolites in feathers (fCORT; in picograms per millimetre or picograms per milligram) in tree swallows (**a**) and sharp-tailed grouse (**b** and **c**). Values in (a) represent a pool of 23 individual tree swallows, whereas values in (b) and (c) are body and flight feathers, respectively, from one individual sharp-tailed grouse. Regardless of methanol/feather concentration, small samples always had higher in picograms per millimetre or picograms per milligram fCORT than larger samples.

If extraction efficiency were causing the small sample artefact, then increasing the concentration of methanol to milligrams or millimetres of feather should: (i) increase the amount of CORT extracted until extraction efficiency saturates (Fig. 2, left panel); and (ii) once that critical concentration is reached, we should detect the same amount of CORT per feather across a wide range of feather weights and lengths (Fig. 2, right panel). However, changing the amount of methanol relative to feather length or mass did not eliminate the small sample artefact. We consistently found that small samples had greater pg/mm or pg/mg than larger samples across a wide range of methanol volumes (Fig. 3). Even when we increased the amount of methanol as high as 2 ml/mm feather (80 ml of methanol for a 40 mm feather), there was still an effect of length. Overall, feather length and mass always had larger effects on picograms of CORT than concentration (β < −10, *P* < 0.001 for length/mass changes in all assays; Table 2). We did find a significant interaction between concentration and mass for one of the three assays, but this appears to be driven by small samples extracted in small amounts of methanol (β_concentration*mass _= −1.18, *P* = 0.03).

**Table 2:** Generalized linear model results of the effects of concentration (mL methanol per mm or mg of feather) and feather amount on picograms per millimetre or picograms per milligram corticsterone

Assay	β ± SE	*t*	*P*-value
Assay 1: tree swallow, 0.1–10 ml/mg			
Intercept (1 mg feather)	26.8 ± 2.02	13.25	<0.0001
5 mg feather	−18.78 ± 2.86	−6.56	<0.0001
Concentration	1.07 ± 0.37	2.94	<0.001
Concentration:mass	−1.18 ± 0.52	−2.27	0.03
Assay 2: sharp-tailed grouse 0.05–0.5 ml/mm methanol			
Intercept (10 mm feather)	19.17 ± 0.85	22.63	<0.0001
20 mm feather	−10.13 ± 0.87	−11.59	<0.0001
40 mm feather	−14.18 ± 0.87	−16.23	<0.001
Concentration	1.12 ± 2.24	0.50	0.62
Assay 3: sharp-tailed grouse 0.1–2 ml/mm			
10 mm feather	24.56 ± 2.21	11.11	<0.0001
20 mm feather	−11.43 ± 2.18	−5.25	0.0001
Concentration	2.75 ± 1.73	1.59	0.135

The amount of feather (length or mass) consistently explained more of the variance in picograms per millimetre or picograms per milligram corticosterone than did concentration.

### Part III: faecal and feather glucocorticoids

**Figure 4:**
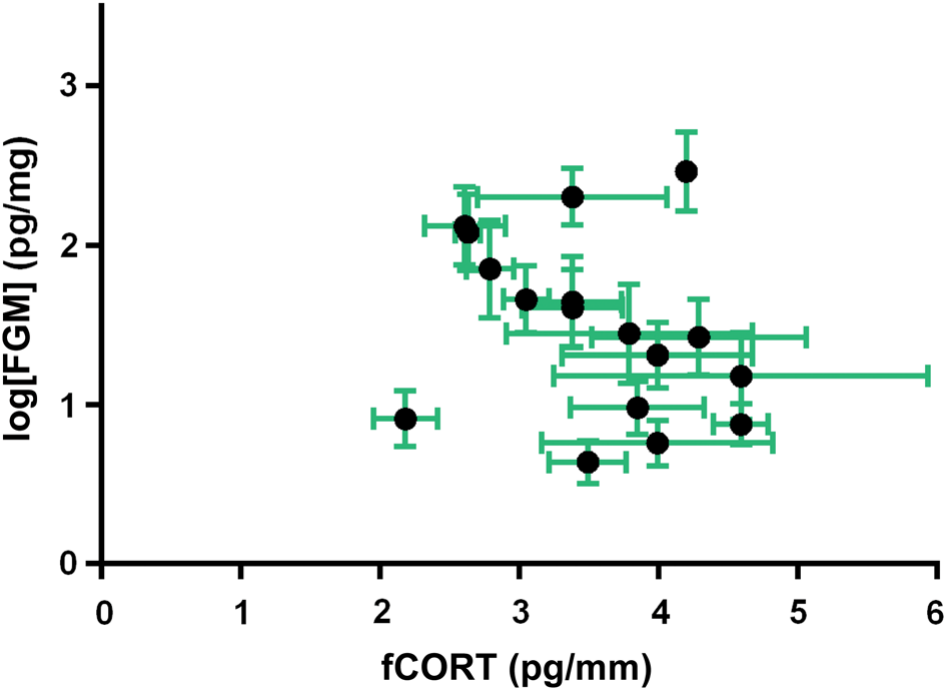
Average feather corticosteroid metabolites (fCORT; in picograms per millimetre) and average faecal glucocorticoid metabolites (FGM; in picograms per milligram) plus standard errors for 17 individual house sparrows.

We found no relationship between fCORT and FGMs in captive house sparrows (95% confidence interval around β [−0.179, 0.331]; Fig. 4 and Table 3). However, there was a strong effect of mass on FGMs (95% confidence interval around β [−0.809, −0.222]).

**Table 3:** Results of a generalized linear model for the effect of feather corticosterone on faecal corticosteroid metabolites

Variable	β ± SE	*t*	*P*-value
Average corticosterone (pg/mm feather)	0.169 ± 0.14	−1.238	0.236
Average (faecal mass)	−0.823 ± 0.14	−6.028	<0.0001

Although we found a strong effect of sample mass on picograms per milligram corticosterone in faeces, feather corticosterone (in picograms per millimetre) was unrelated to faecal corticosterone (in picograms per milligram).

### Part IV: high-performance liquid chromatography

**Figure 5:**
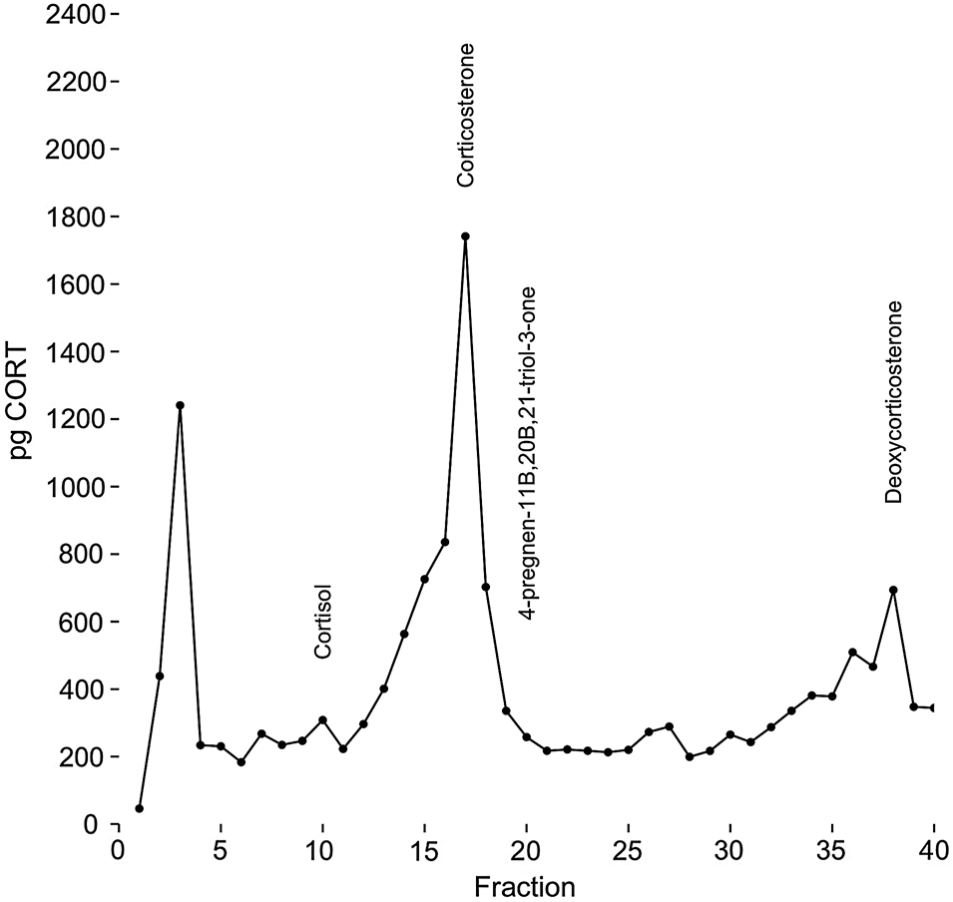
High-performance liquid chromotographic analysis of feather samples from sharp-tailed grouse. Points represent immunoreactivity (in picograms of corticosterone per fraction), and elution positions of standards are noted where appropriate. All standards are not included in this figure, and they are included only if they correspond to or occur near large peaks of immunoreactivity or have been identified in feathers in the past (cortisol).

Our antibody detected a main immunoreactive peak at fraction 17, consistent with our corticosterone standard. However, this peak was broad and appeared to be elevated over background from fractions 14 to 18. Our only other significant peak coincided with deoxycorticsterone (a CORT precursor), in fraction 38 (Fig. 5). We also detected an unknown peak very early (fraction 3) that did not correspond to any of the standards used in our analysis.

## Discussion

The fCORT is increasingly measured in a variety of species because of the ease of feather collection and the potential insight offered by integrated hormone levels measured in the feather (in comparison to the brief window offered by plasma samples). Recent papers focus on using fCORT for conservation aims or to assess physiology when animals are difficult to capture (e.g. Fairhurst *et al.*, 2015; Rebolo-Ifrán *et al.*, 2015; Strong *et al.*, 2015; Latta *et al.*, 2016; Lodjak *et al.*, 2016). Understanding how environmental factors affect organismal physiology and performance is a crucial goal for the study of conservation physiology in our changing world. Feather CORT is a useful tool for this aim; however, we must be cautious to validate techniques fully before interpreting results (Sheriff *et al.*, 2011). We hoped to explore significant methodological issues to allow for more consistent and informative data collection in this field. We found that the small sample artefact is consistently present across species and feather types, with small samples reading artificially high amounts of fCORT per milligram and per millimetre. Unfortunately, changing the amount of methanol relative to feather volume did not eliminate this effect. Furthermore, we found no relationship between concurrent measurements of faecal and fCORT. Lastly, we found that the Sigma-Aldrich antibody is primarily detecting corticosterone and a corticosterone precursor, deoxycorticosterone, although there may be limited cross-reactivity with a progesterone metabolite as well as an unknown compound.

The small sample artefact has been a consistent problem in the faecal CORT literature (Millspaugh and Washburn, 2004; Tempel and Guitierrez, 2004; Cyr and Romero, 2008). In mammals, changing the amount of solvent relative to faecal matter (and other methodological modifications) seemed to correct the problem (Hayward *et al.*, 2010). Our failure to correct for small samples by increasing the amount of methanol during extraction leads to several possibilities. First, it may be that our scale produced biased measurement error at small masses. Hayward *et al*. (2010) found that using a more accurate scale improved their measurement of CORT in small samples. However, we find that the small sample artefact still persists when using a more accurate scale in our laboratory (T. Kelly and C. Breuner, personal communication), although we have not compared measurements from several scales on the same sample. Future work should examine scale accuracy as a potential cause of the small sample artefact. Second, while we found that the Sigma-Aldrich antibody is mostly measuring corticosterone, there is still significant cross-reactivity with both identified (deoxycorticosterone) and unidentified metabolites. Other studies on corticosterone deposition in chicken eggs have found similar large early immunoreactive peaks that do not correspond to any hormones or metabolites that are identifiable by HPLC (Rettenbacher *et al.*, 2009). Although cortisol has been identified in feathers by previous work, we confirmed that the Sigma-Aldrich antibody is likely not to be measuring cortisol (Koren *et al.*, 2012). Although other laboratories have demonstrated parallelism in measurement of corticosterone with the Sigma-Aldrich antibody, none has explored parallelism of other corticosterone metabolites (Bortolotti *et al.*, 2008). Imperfect parallelism of the antibody with other metabolites would disproportionately affect small samples, because we may be consistently over-predicting the amount of hormone in smaller samples while under-predicting the amount of hormone in large samples.

In the past, researchers have sought to validate fCORT by comparing endogenous plasma corticosterone concentrations with fCORT within individuals and have obtained mixed results that seem driven by outliers or experimental corticosterone treatment (Bortolotti *et al.*, 2008; Fairhurst *et al.*, 2013; Jenni-Eiermann *et al.*, 2015). In these cases, it appears that plasma corticosterone predicts fCORT when corticosterone implants are given, but is not a good predictor using plasma corticosterone concentrations alone. We thought that because plasma sampling represents such a short time period compared with the duration of feather growth, using another integrated measure of corticosterone physiology would prove more informative in this context. Our lack of correlation may result from one of several possibilities. First, fCORT may not represent circulating corticosterone concentrations over time. However, it is also possible that there is differential metabolism of corticosterone in the gut and feather follicle; our antibody is likely not to be equally specific for all metabolites and would therefore recognize different amounts in the two measures. It may also be a result of inaccuracies in the measurement of fCORT. Recent work using radioactively labelled corticosterone has shown that endogenous corticosterone is deposited in the feather, but we do not yet know what affects the rate of corticosterone uptake into the feather (Jenni-Eiermann *et al.*, 2015). In plasma, corticosterone is bound to corticosteroid-binding globulin (CBG), which regulates the availability of corticosterone to tissues (Breuner and Orchinik, 2002; Breuner *et al.*, 2013). If only unbound (free) corticosterone can be deposited into feathers, then challenge-induced changes in CBG may alter the corticosterone deposition rate. These types of effects may influence our estimates of fCORT in unpredictable ways, especially given that there is some evidence for local regulation of CBG by specific tissues (reviewed by Malisch and Breuner, 2010). To date, no research has examined the relationships between free corticosterone or CBG and fCORT, and this might be an important avenue to allow us to gain a better understanding of the dynamic relationships between plasma corticosterone and fCORT. Finally, there is evidence of local synthesis of corticosterone within the skin, which could mean that fCORT is a mix of local and global corticosterone secretion and synthesis, and this process could obscure any expected correlation between faecal and feather glucorticoids (Taves *et al.*, 2011).

Although we see reliable increases in fCORT in response to supraphysiological exogenous concentrations, in order to make inferences in wild populations using fCORT we should seek to unravel the relationships between natural stressors and corticosterone deposition into feathers using laboratory and wild birds (Fairhurst *et al.*, 2013; Jenni-Eiermann *et al.*, 2015). Future work should focus on measuring fCORT in animals exposed to potent stressors, such as food deprivation, because these effects appear to be species specific. For example, food-deprived caspian tern chicks showed increased plasma corticosterone but also reduced their feather growth rate and therefore deposited less corticosterone into their feathers (Patterson *et al.*, 2015). This result at first seems counterintuitive, as previous authors have suggested that slower-growing feathers might be exposed to greater amounts of circulating corticosterone (Bortolotti *et al.*, 2009). At the very least, Patterson *et al.* (2015) demonstrate that relationships between external challenge, feather growth rate and corticosterone deposition are complex and do not always mirror plasma corticosterone fluctuations.

The goal of conservation physiology is to understand how animals react physiologically to changing environments and, eventually, to mediate these effects through intervention without causing further harm (Wikelski and Cooke, 2006). Measurements of fCORT cannot be applied to these aims without thorough validation using stressors that we regularly expect to affect plasma corticosterone. For example, although we might expect fCORT to be more sensitive to perturbations such as low-level contaminant exposure or anthropogenic disturbance because of its integration of hormone concentrations over long time periods, it is still impossible to interpret results in natural systems until we validate fCORT in situations that we know to change corticosterone concentrations consistently in the species of interest. Without this type of thorough validation, any observed differences might be the result of problems associated with the technique and not with the biology of natural systems.

In fCORT studies, it is common to use an adrenocorticotrophic hormone challenge to assess the validity of an assay for a given species. However, this technique is not possible in feathers because adrenocorticotrophic hormone challenges lead to increases in corticosterone for only a few hours post-injection (Wasser *et al.*, 2000), and feathers grow over days to weeks. Finding a similar, biologically relevant validation test should be a priority for fCORT research, in order to eliminate potential between-species differences and assess the appropriateness of the assay before making inferences. Researchers should continue to correlate fCORT with relevant measures of condition, anthropogenic disturbance or performance across species.

Overall, we recommend that researchers continue to evaluate the use of fCORT measures stringently in their own systems. It seems that until we can uncover the causes of the small sample artefact, researchers should standardize the amount of feather material across samples, as recommended by Lattin *et al*. (2011). Although we obtained similar results to Lattin *et al*. (2011) showing that there is no relationship between extracted fCORT and feather mass above 20 mg, it does not seem necessary or feasible to recommend that researchers collect samples larger than this for analysis of fCORT. Twenty milligrams of feathers from a small bird can be as many as 35–50 feathers, and this is unfeasible, especially in endangered or threatened species that are of particular interest to the conservation physiologist. As we found very little variation in fCORT extracted in feathers of the same mass during our methanol extraction experiments, it seems acceptable to analyse small feather samples so long as researchers take care to standardize the amount of feather material in each sample. It may also be beneficial to test other antibodies using similar HPLC methods to determine whether there are viable alternatives for this assay. However, researchers in other laboratories have reported limited success using other antibodies (L. M. Romero, personal communication). Another alternative is to eliminate the problem of antibody specificity altogether by using ultra-high-performance liquid chromatography–tandem mass spectrometry (LCMS) to measure CORT and its metabolites more precisely. Although one study found that results were comparable between enzyme immunoassay and LCMS (Jenni-Eiermann *et al*., 2015), they did not perform this analysis using the Sigma-Aldrich antibody, which is the most widely used to measure fCORT. Analyses of fCORT using LCMS in an ecological context did find links between fCORT and survival in house sparrows (Koren *et al.*, 2012). Similar mass spectrometry or gas chromatography techniques are relatively common for the analysis of corticosterone in hair, although similar issues persist in this field as well (Gao *et al.*, 2013).

We believe that feather corticosterone is potentially a useful measure of stress physiology in wild populations, but authors should be cautious regarding their interpretation of the results until more validation work is available.

## Funding

This work was supported by the National Science Foundation (PSI-0747361 to C.W.B.), a Jerry O’Neal National Park Fellowship Grant to W.K.H. and the Glacier National Park Conservancy.
